# Risk of Adverse Vascular Events in Newly Diagnosed Glioblastoma Multiforme Patients Treated with Bevacizumab: a Systematic Review and Meta-Analysis

**DOI:** 10.1038/srep14698

**Published:** 2015-10-01

**Authors:** Xiaoqing Li, Rongzhong Huang, Zhongye Xu

**Affiliations:** 1 Cancer Research Institute of Chongqing, Chongqing Cancer Hospital, 181 Hanyu Road, Shapingba, Chongqing 400030, China; 2 Department of Neurosurgery, the Second Affiliated Hospital of Chongqing Medical University, No. 76 Linjiang Road, Chongqing 400010, China; 3 Department of Rehabilitation Medicine, the Second Affiliated Hospital of Chongqing Medical University, No. 76 Linjiang Road, Chongqing 400010, China

## Abstract

Previous evidence suggests that the humanized anti-VEGF antibody bevacizumab increases thrombosis risk in glioma patients. Here, we comprehensively assessed the risk of adverse vascular events in adult glioma patients receiving bevacizumab therapy. Systematic searches of MEDLINE, EMBASE, and the Cochrane Library were conducted to find prospective phase II/III clinical trials on adult bevacizumab-treated glioma patients and non-bevacizumab-treated controls that reported data on adverse vascular events. Four high-quality trials were finally included in the systematic review, scoring greater than or equal to 7/8 on the Newcastle-Ottawa Scale. Three trials provided sufficient data for four meta-analytical comparisons between bevacizumab-treated and control groups of newly diagnosed glioblastoma multiforme (GBM) patients: all-cause discontinuation, thrombocytopenia, deep vein thrombosis (DVT), and pulmonary embolism. None of these adverse outcomes were found to be significantly different between bevacizumab-treated and control groups (*P* > 0.05); however, there was a trend toward significance with regard to bevacizumab therapy and the risk of pulmonary embolism (*P* = 0.07). As there was a trend toward significance with regard to bevacizumab therapy and the risk of pulmonary embolism, anticoagulation may be advisable in certain newly diagnosed adult GBM patients who display a history of thromboembolism and/or more serious risk factors for thromboembolic events.

Vascular endothelial growth factor (VEGF) plays a key role in tumor growth, invasion, and metastasis through promoting tumor angiogenesis^1^. Bevacizumab was developed as a humanized monoclonal antibody against VEGF and has been shown to be effective for a variety of solid malignancies, including colorectal cancer, breast cancer, ovarian cancer, renal cancer, non-small-cell lung cancer, and glioma^1^. Because of VEGF’s key role in vascular function and angiogenesis, bevacizumab therapy has been observed to produce serious adverse vascular events, including bleeding, thromboembolic events, and neutropenia^1^. A 2011 meta-analysis by Ranpura *et al.* consisting of 16 RCTs including 10,217 patients with a variety of advanced solid tumors revealed that bevacizumab was associated with a higher risk of fatal bleeding but showed no association with fatal adverse events secondary to neutropenia, pulmonary embolism, or stroke^1^.

In particular, bevacizumab appears to increase thrombosis risk in glioma patients, whom are already at increased risk of thromboembolic events with rates approaching 30% in patients with high-grade glioma^2^. This effect has been attributed to a combination of multiple factors, including a tumor-induced hypercoagulable state, neurological deficits, immobilization, and steroid use^3^. A 2014 meta-analysis by Simonetti *et al.* consisting of 2,208 malignant glioma patients revealed that bevacizumab therapy is associated with a significantly increased risk of venous thromboembolism (VTE)^4^.

Despite this evidence of increased VTE risk in bevacizumab-treated glioma patients from Simonetti *et al.*’s work, there has been no meta-analysis that has comprehensively analyzed the risk of adverse vascular events in glioma patients undergoing bevacizumab therapy beyond VTE risk. Therefore, the aim of this systematic review and meta-analysis will be to comprehensively assess the risk of cerebrovascular and other adverse vascular events in adult glioma patients receiving bevacizumab therapy.

## Materials and Methods

### Search Strategy

A systematic review and meta-analysis was conducted according to the Preferred Reporting Items for Systematic Reviews and Meta-Analyses (PRISMA) guidelines^5^. Relevant trials were identified from systematic searches of three major electronic databases (MEDLINE, EMBASE, and the Cochrane Library) up to December 2014 with different combinations of the following key words: (glioma OR astrocytoma OR glioblastoma OR GBM OR oligodendroglioma OR ependymoma) AND (avastin OR bevacizumab) AND (vascular OR hemorrhag* OR ICH OR SAH OR ischem* OR cerebrovascular OR CVA OR stroke OR thrombo* OR embol*). Additional relevant articles were obtained by scanning reference lists of the articles identified in the initial searches. No language restrictions were imposed during the initial searches.

### Study Selection

The purpose of this study was to determine whether bevacizumab increases the risk of cerebrovascular and other adverse vascular events in adult patients with glioma. Therefore, we only selected (i) prospective phase II/III clinical trials including (ii) adult patients with glioma treated with bevacizumab and (iii) non-bevacizumab-treated controls that (iv) reported data on cerebrovascular and other adverse vascular events (e.g., hemorrhagic stroke, ischemic stroke, deep vein thrombosis (DVT), pulmonary embolism, etc.).

Reports limited to non-general populations (e.g., young adults, elderly) and studies failing to report adverse vascular events were excluded. If single-center reports were compiled into larger, multicenter series, single-site data were not included unless unique patients could be identified. Non-English studies, non-Chinese studies, Phase I studies, retrospective studies, animal studies, pediatric studies, case reports, reviews, editorials/commentaries, conference abstracts/summaries, and technical reports were all excluded.

### Data Extraction

Data extraction was conducted independently by two co-authors, and any discrepancies between these co-authors were resolved by discussion and consensus. For each study, we extracted the following information: first author’s name, year of publication, country of publication, number of included patients, study arms, bevacizumab dose (mg/kg), additional therapies and dose (if any) (e.g., irinotecan, temozolomide, fotemustine), radiation therapy and total dose (Gy), median follow-up period, proportion of protocol violations (%), rates of all-cause discontinuation (%), and rates of adverse vascular events (%), including thrombocytopenia, angina pectoris, myocardial infraction (MI), transient ischemic attacks (TIA), ischemic stroke, hemorrhage stroke, DVT, pulmonary embolism (PE), other vascular events (e.g., esophageal hemorrhage, gastric hemorrhage), and vascular-related mortality.

### Methodological Quality Assessment

We assessed the methodological quality of all included studies by the Newcastle-Ottawa Scale (NOS)^6^. A quality score, with a maximum score of nine points, was calculated from three major NOS components: (i) group selection, (ii) comparability, and (iii) assessment of outcome or exposure.

### Statistical Analysis

STATA 12.0 (STATA Corp., College Station, TX, USA) and the Cochrane Collaboration Review Manager 5 (RevMan 5) were used for the statistical analysis^7^. A two-tailed *P*-value < 0.05 was considered statistically significant. For the calculation of incidence, the number of patients with each adverse vascular event and the number of bevacizumab-treated patients were extracted from the safety profiles of the included studies. The proportion of patients with each adverse vascular event and the 95% CIs were then derived from each trial. Statistical heterogeneity among the included trials was quantified with the I^2^ statistic (100%*[Q-df]/Q), which estimates the percentage of total variation across studies attributable to heterogeneity over chance^8^. If the I^2^ value was greater than 50%, the assumption of homogeneity was deemed invalid, and the random-effects model was used; otherwise, the fixed-effects model was used^9^. Publication bias was evaluated with visual inspection of funnel plots followed by Egger’s test if necessary^10^.

## Results

**Figure 1:**
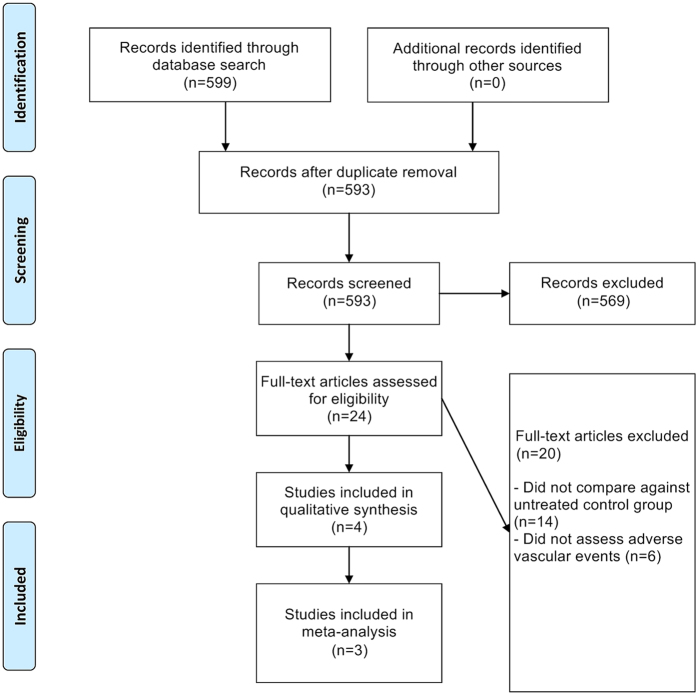
Flowchart of Study Selection.

The flowchart for study selection is provided in Fig. 1. From an initial set of 599 records, a total of four trials were finally included in the systematic review^11^,^12^,^13^,^14^. All four studies consisted of GBM patients. The detailed characteristics of the four included studies are provided in Table 1, and the NOS quality assessment of these included studies is provided in Table 2. All four studies were of relatively high-quality, scoring greater than or equal to 7/8 on the NOS.

**Figure 2:**
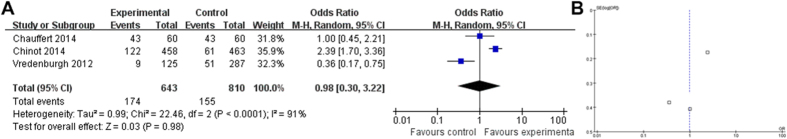
Meta-Analysis of All-Cause Discontinuation. (**A**) Forest plot and (**B**) funnel plot.

**Figure 3:**
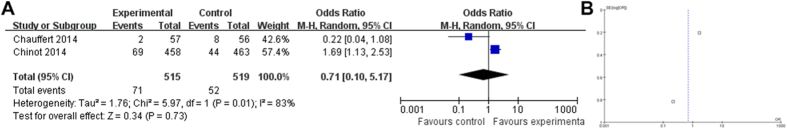
Meta-Analysis of Thrombocytopenia. (**A**) Forest plot and (**B**) funnel plot.

**Figure 4:**
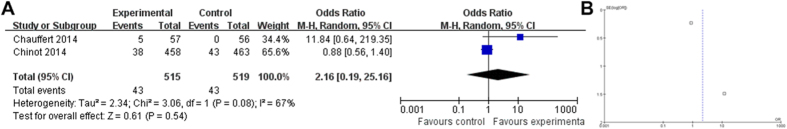
Meta-Analysis of Deep Vein Thrombosis. (**A**) Forest plot and (**B**) funnel plot.

**Figure 5:**
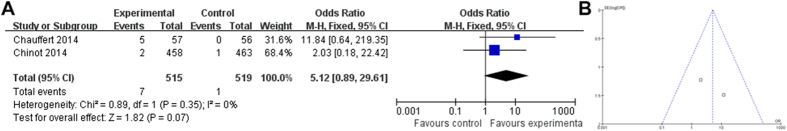
Meta-Analysis of Pulmonary Embolism. (**A**) Forest plot and (**B**) funnel plot.

Of these four trials, three trials provided sufficient data for statistical control-based comparisons and were included in the quantitative meta-analysis^11^,^12^,^14^. From these trials, four meta-analytical comparisons between bevacizumab-treated and control groups were possible: all-cause discontinuation (Fig. 2), thrombocytopenia (Fig. 3), DVT (Fig. 4), and pulmonary embolism (Fig. 5). Notably, none of these adverse outcomes were found to be significantly different between bevacizumab-treated and control groups (*P* > 0.05; Figs 2A–5A). However, there was a trend toward significance with regard to bevacizumab therapy and the risk of pulmonary embolism (*P* = 0.07). There was significant heterogeneity (I^2^ > 50%) in the comparisons of all-cause discontinuation (Fig. 2A), thrombocytopenia (Fig. 3A), and DVT (Fig. 4A). However, the comparison of pulmonary embolism showed no detectable heterogeneity (I^2^ = 0%, *P* = 0.35; Fig. 5A). Inspection of the funnel plots revealed no apparent publication bias (Figs 2B–5B).

## Discussion

Here, we found that bevacizumab therapy does not significantly affect the risk of all-cause discontinuation, thrombocytopenia, DVT, or pulmonary embolism in newly diagnosed adult GBM patients (*P* > 0.05; Figs 2A–5A). However, there was a trend toward significance with regard to bevacizumab therapy and the risk of pulmonary embolism (*P* = 0.07, Fig. 5A).

Gliomas, such as GBM, originate from neural stem cells, neural progenitor cells, or de-differentiated mature neural cells in the brain matter and are graded on the basis of several histopathological factors (i.e., tumor cell differentiation, cellularity, cytonuclear atypia, mitotic activity, microvascular proliferation, and necrosis) using the World Health Organization grading scheme (WHO I-IV): WHO grade II (diffuse infiltrating low-grade astrocytomas/oligodendrogliomas), WHO grade III (anaplastic astrocytomas/oligodendrogliomas), or WHO grade IV (high-grade GBM)^15^,^16^. As high-grade malignant gliomas are hypoxic and highly vascularized, these tumors express relatively high VEGF levels that positively correlates with aggressiveness, making VEGF a promising therapeutic target^16^. Bevacizumab was developed to target VEGF with high affinity and specificity and serves to directly inhibit VEGF-associated angiogenic effects by blocking VEGF receptor (VEGF-R) activation^16^. Bevacizumab demonstrates a 30–50% response rate, administered alone or in combination with irinotecan, with a 35–50% estimated six-month progression-free survival for recurrent glioblastoma^15^.

Two large clinical studies (AVAglio and RTOG 0825^12^,^17^) have attributed several toxicities to bevacizumab therapy, including hypertension (10.3%), proteinuria (3.7%), poor wound healing (1.5%), and thromboembolic events (4.1% arterial, 7.3% venous)^16^. Moreover, a previous multivariate analysis found that recent surgery (22-fold higher), malignant neoplasm (with (six-fold higher) or without chemotherapy (four-fold higher)), and neurologic disease with extremity paresis (three-fold) are independent risk factors for VTE^18^. As this previous evidence suggests that bevacizumab treatment and several factors associated with glioma status (i.e., recent cranial surgery, malignant neoplasm, chemotherapy, etc.) appear to increase the risk of VTE^16^, we hypothesized that bevacizumab therapy would have negative effects upon adverse vascular events in glioma patients receiving bevacizumab therapy.

However, our current findings revealed that bevacizumab therapy does not significantly affect the risk of all-cause discontinuation, thrombocytopenia, DVT, or pulmonary embolism in newly diagnosed adult GBM patients (*P* > 0.05; Figs 2A–5A). However, there was a trend toward significance with regard to bevacizumab therapy and the risk of pulmonary embolism (*P* = 0.07, Fig. 5A). Our findings are consistent with a comprehensive review of three National Cancer Institute (NCI) phase II bevacizumab trials (NCT00271609, NCT00586508, and NCT00667394) that reported unremarkable thrombosis rates in 210 recurrent malignant glioma cases receiving bevacizumab therapy^19^. Moreover, our results largely coincide with those of RTOG 0825, which revealed approximately equivalent risk in thrombocytopenia between the bevacizumab group vs. the placebo group (11.1% vs. 11.7%) but slightly increased prevalence of thromboembolic disease in the bevacizumab group vs. the placebo group (7.7% vs. 4.7%)^17^.

In terms of treatment recommendations going forward, anticoagulation therapy has been previously used during bevacizumab treatment, especially in those who display a history of thromboembolism and/or more serious risk factors for thromboembolic events^18^. Although anticoagulation should theoretically increase the risk of hemorrhagic events, the overall rate of hemorrhagic complications has not been reported to be significantly higher in bevacizumab-treated glioma patients receiving anticoagulant therapy^16^,^20^. Therefore, although our findings do not support a statistically significant increased risk of all-cause discontinuation, thrombocytopenia, DVT, or pulmonary embolism in newly diagnosed adult GBM patients (*P* > 0.05; Figs 2A–5A), we still found a trend toward significance with regard to bevacizumab therapy and the risk of pulmonary embolism (*P* = 0.07, Fig. 5A). Thus, anticoagulation may still be advisable in particular adult GBM patients who display a history of thromboembolism and/or more serious risk factors for thromboembolic events.

In order to assess this risk of thromboembolic events in adult glioma patients, several studies have provided guidance with respect to prognostic biomarkers for thromboembolic risk in this patient population. For example, a recent prospective study by Thaler *et al.* investigated 11 potential biomarkers for predicting VTE risk in 144 newly diagnosed adult high grade glioma patients found significant associations between future VTE risk and leukocyte count, platelet count, sP-selectin, prothrombin fragment 1 + 2 (F 1 + 2), FVIII activity, and D-dimer^21^. Another study by Ay *et al.* showed that elevated D-dimer and F 1 + 2 could stratify adult glioma patients prone to developing VTE^22^. Jenkins *et al.* has suggested a prediction model that combines circulating D-dimer, F 1 + 2, VEGF or plasminogen activator inhibitor-1 (PAI-1) levels, as well as tumoral tissue factor (TF) expression to risk stratify for VTE in adult glioma patients^23^.

There are several limitations to this study. First, although we searched for multiple adverse vascular outcomes during data extraction, we were unable to find data on angina pectoris, MI, TIA, hemorrhagic stroke, ischemic stroke, other vascular events (e.g., esophageal hemorrhage, gastric hemorrhage, etc.), or vascular-related mortality. Second, the included studies did not distinguish distal from proximal DVT when reporting DVT outcomes. Third, the ability to accurately detect the reported outcomes may have varied among the study centers, resulting in bias of the reported incidence rates. Fourth, although all the included studies applied conventional temozolomide and radiotherapy in conjunction with bevacizumab treatment, the Chauffert 2014 study also used irinotecan in both the experimental and control groups, which may contributed to the heterogeneity in some of the comparisons. Fifth, significant heterogeneity (I^2^ > 50%) was found in the comparisons of all-cause discontinuation (Fig. 2A), thrombocytopenia (Fig. 3A), and DVT (Fig. 4A). Finally, relevant confounding factors that have been recognized to affect the risk of adverse vascular events at the patient level—such as patient age, gender, obesity, steroid use, and smoking history^24^—could not be assessed in this study-level meta-analysis. Future trials analyzing the risks of bevacizumab therapy on glioma patients should be certain to analyze and report on these confounding factors.

In conclusion, bevacizumab therapy does not appear to significantly affect the risk of all-cause discontinuation, thrombocytopenia, DVT, or pulmonary embolism in newly diagnosed adult GBM patients. However, there was a trend toward significance with regard to bevacizumab therapy and the risk of pulmonary embolism. Thus, anticoagulation may be advisable in certain newly diagnosed adult GBM patients who display a history of thromboembolism and/or more serious risk factors for thromboembolic events. Further large-scale randomized, controlled trials are needed to assess bevacizumab’s effects on adverse vascular events in adult glioma patients.

## Additional Information

**How to cite this article**: Li, X. *et al.* Risk of Adverse Vascular Events in Newly Diagnosed Glioblastoma Multiforme Patients Treated with Bevacizumab: a Systematic Review and Meta-Analysis. *Sci. Rep.* **5**, 14698; doi: 10.1038/srep14698 (2015).

## Figures and Tables

**Table 1:** Characteristics of Included Trials.

**Study**	**Country**	**Number (n)**		**Mean age (years)**		**Gender (n, M/F)**		**Type of glioma**	Bevacizumab dose, mg/kg* **(+ other chemotherapy)**		**Radiation therapy, total dose (Gy)**		**Protocol violations**		**Median follow-up**
		**B**	**C**	**B**	**C**	**B**	**C**		**B**	**C**	**B**	**C**	**B**	**C**	
Chauffert 2014	France	60	60	60.2	60.9	34/26	37/23	GBM	10 (+IRI-125+TMZ-75)	0 (+IRI-125+TMZ-75)	60	60	3/60	3/60	11.1 month
Chinot 2014	France	458	463	57	56	282/176	298/165	GBM	10 (+TMZ-75)	0 (+TMZ-75)	60	60	3/458	1/463	14.4 months (bevacizumab group); 13.7 months (control group)
Clarke 2014	USA	59	133	54	56	—	—	GBM	10 (+ERL-150-200 + TMZ-75)	0 (+TMZ-75)	60	60	0/59	—	—
Vredenburgh 2012	USA	125	287	56.2	—	74/51	—	GBM	10 (+TMZ-75)	0 (+TMZ-75)	59.4	60	0/32	—	21 months

Abbreviations: B, bevacizumab-treated group; C, control group; GBM, glioblastoma multiforme; IRI-125, inrinotecan at 125 mg/m^2^ every two weeks for four cycles; TMZ-75, temozolomide at 75 mg/m^2^ daily; ERL-150-200, erlotinib 150–200 mg daily.

^*^Standard dosing of bevacizumab given at 10 mg/kg every two weeks.

**Table 2:** Methodological Quality Assessment by the Newcastle-Ottawa Scale (NOS)

**Study**	**Selection**				**Comparability**	**Outcome or Exposure**			**Scores**
	**1**	**2**	**3**	**4**	**1**	**1**	**2**	**3**	
Chauffert 2014	*	*	*	*	*	*	*	*	8
Chinot 2014	*	*	*	*	*	*	*	*	8
Clarke 2014	*	—	*	*	*	*	*	*	7
Vredenburgh 2012	*	*	*	*	*	*	*	*	8

Note: Each asterisk (*) denotes one point on the NOS.

## References

[b1] Ranpura V., Hapani S. & Wu S. Treatment-related mortality with bevacizumab in cancer patients: a meta-analysis. Jama 305, 487–494 (2011). 21285426 10.1001/jama.2011.51

[b2] Jo J., Schiff D. & Perry J. Thrombosis in brain tumors. Seminars in thrombosis and hemostasis 40, 325 (2014). 24599439 10.1055/s-0034-1370791

[b3] Stupp R., Brada M., van den Bent M., Tonn J.-C. & Pentheroudakis G. High-grade glioma: ESMO Clinical Practice Guidelines for diagnosis, treatment and follow-up. Annals of Oncology, mdu050, 10.1093/annonc/mdu050 (2014). 20555079

[b4] Simonetti G. *et al.* Safety of bevacizumab in patients with malignant gliomas: a systematic review. Neurological Sciences 35, 83–89 (2014). 24281944 10.1007/s10072-013-1583-6

[b5] Moher D., Liberati A., Tetzlaff J. & Altman D. G. Preferred reporting items for systematic reviews and meta-analyses: the PRISMA statement. Annals of internal medicine 151, 264–269 (2009). 19622511 10.7326/0003-4819-151-4-200908180-00135

[b6] Stang A. Critical evaluation of the Newcastle-Ottawa scale for the assessment of the quality of nonrandomized studies in meta-analyses. European journal of epidemiology 25, 603–605 (2010). 20652370 10.1007/s10654-010-9491-z

[b7] Borenstein M., Hedges L. V., Higgins J. P. & Rothstein H. R. Introduction to meta-analysis. (John Wiley & Sons, 2011).

[b8] Higgins J. P., Thompson S. G., Deeks J. J. & Altman D. G. Measuring inconsistency in meta-analyses. BMJ: British Medical Journal 327, 557 (2003). 12958120 10.1136/bmj.327.7414.557 PMC192859

[b9] Shadish W. R. & Haddock C. K. Combining estimates of effect size. The handbook of research synthesis and meta-analysis 2, 257–277 (2009).

[b10] Peters J. L., Sutton A. J., Jones D. R., Abrams K. R. & Rushton L. Comparison of two methods to detect publication bias in meta-analysis. Jama 295, 676–680 (2006). 16467236 10.1001/jama.295.6.676

[b11] Chauffert B. *et al.* Randomized phase II trial of irinotecan and bevacizumab as neo-adjuvant and adjuvant to temozolomide-based chemoradiation compared with temozolomide-chemoradiation for unresectable glioblastoma: final results of the TEMAVIR study from ANOCEF. Annals of Oncology 25, 1442–1447 (2014). 24723487 10.1093/annonc/mdu148

[b12] Chinot O. L. *et al.* Bevacizumab plus radiotherapy–temozolomide for newly diagnosed glioblastoma. New England Journal of Medicine 370, 709–722 (2014). 24552318 10.1056/NEJMoa1308345

[b13] Clarke J. L. *et al.* A single-institution phase II trial of radiation, temozolomide, erlotinib, and bevacizumab for initial treatment of glioblastoma. Neuro-oncology 16, 984–990 (2014). 24637230 10.1093/neuonc/nou029 PMC4057142

[b14] Vredenburgh J. J. *et al.* Addition of bevacizumab to standard radiation therapy and daily temozolomide is associated with minimal toxicity in newly diagnosed glioblastoma multiforme. International Journal of Radiation Oncology* Biology* Physics 82, 58–66 (2012). 10.1016/j.ijrobp.2010.08.058 21036490

[b15] Ricard D. *et al.* Primary brain tumours in adults. The Lancet 379, 1984–1996 (2012). 10.1016/S0140-6736(11)61346-9 22510398

[b16] Khasraw M., Ameratunga M. & Grommes C. Bevacizumab for the treatment of high-grade glioma: an update after Phase III trials. Expert opinion on biological therapy 14, 729–740 (2014). 24655021 10.1517/14712598.2014.898060

[b17] Gilbert M. R. *et al.* A randomized trial of bevacizumab for newly diagnosed glioblastoma. New England Journal of Medicine 370, 699–708 (2014). 24552317 10.1056/NEJMoa1308573 PMC4201043

[b18] Heit J. A. *et al.* Risk factors for deep vein thrombosis and pulmonary embolism: a population-based case-control study. Archives of internal medicine 160, 809–815 (2000). 10737280 10.1001/archinte.160.6.809

[b19] Odia Y., Shih J. H., Kreisl T. N. & Fine H. A. Bevacizumab-related toxicities in the National Cancer Institute malignant glioma trial cohort. Journal of neuro-oncology 120, 431–440 (2014). 25098701 10.1007/s11060-014-1571-6

[b20] Norden A. D. *et al.* Safety of concurrent bevacizumab therapy and anticoagulation in glioma patients. Journal of neuro-oncology 106, 121–125 (2012). 21706358 10.1007/s11060-011-0642-1

[b21] Thaler J. *et al.* Biomarkers predictive of venous thromboembolism in patients with newly diagnosed high-grade gliomas. Neuro-oncology, nou106, 10.1093/neuonc/nou106 (2014). PMC4232082 24987133

[b22] Ay C. *et al.* D-dimer and prothrombin fragment 1 + 2 predict venous thromboembolism in patients with cancer: results from the Vienna Cancer and Thrombosis Study. Journal of Clinical Oncology 27, 4124–4129 (2009). 19636003 10.1200/JCO.2008.21.7752

[b23] Jenkins E., Schiff D., Mackman N. & Key N. Venous thromboembolism in malignant gliomas. Journal of Thrombosis and Haemostasis 8, 221–227 (2010). 19912518 10.1111/j.1538-7836.2009.03690.x PMC2834309

[b24] Nightingale A. *et al.* The effects of age, body mass index, smoking and general health on the risk of venous thromboembolism in users of combined oral contraceptives. The European journal of contraception & reproductive health care: the official journal of the European Society of Contraception 5, 265 (2000). 10.1080/13625180008500402 11245554

